# AMPK Knockout Impairs the Formation of Three-Dimensional Spheroids

**DOI:** 10.3390/life15040525

**Published:** 2025-03-22

**Authors:** Yea-In Park, Rackhyun Park, Siyun Lee, Chunghyeon Lee, Inkyu Yoo, Hakhyun Ka, Yang Hoon Huh, Jongkwang Hong, Junsoo Park

**Affiliations:** 1 Division of Biological Science and Technology, Yonsei University, Wonju 26493, Republic of Korea; 2 Department of Life Science, Yong-In University, Yongin 17092, Republic of Korea; 3 Electron Microscopy Research Center, Korea Basic Science Institute (KBSI), Cheongju 28119, Republic of Korea

**Keywords:** AMPK, knockout, 3D culture, spheroid, lysosome

## Abstract

AMP-activated protein kinase (AMPK) is an important regulator of cellular energy homeostasis, and AMPK contributes to cell growth, apoptosis, and autophagy. Although most cell studies have been performed using two-dimensional (2D) cell culture, recent studies have demonstrated that the three-dimensional (3D) spheroid technique is helpful in various cell research fields, such as tumor biology, due to its resemblance to the 3D tissue structure. However, the role of AMPK in 3D spheroid formation has not been characterized clearly. This study used the AMPK knockout cell line to examine the role of AMPK in 3D spheroid formation and is the first report describing the generation of 3D spheroids using AMPK knockout cells. While control cells produced round spheroids with a similar length-to-width ratio, AMPK knockout produced an oval shape with a more significant length-to-width ratio. We demonstrate that AMPK knockout spheroids contain significantly more prominent lysosomes in each cell, indicating that autophagic flux is impaired in 3D spheroids. Finally, flow cytometry analysis showed that AMPK knockout spheroids contain more apoptotic cells than control cells. These results indicate that AMPK is required for efficient 3D spheroid formation.

## 1. Introduction

AMP-activated protein kinase (AMPK) was initially identified as an energy-sensing molecule that senses the AMP-ATP ratio or the ADP-ATP ratio [1,2]. Therefore, AMPK recognizes the energy shortage in the cell, and the activation of AMPK activates the catabolic pathway to produce ATP [3,4]. AMPK activation inhibits the synthesis of fatty acid and cholesterol to reduce energy expenditure by downregulating the adipogenesis genes [5,6]. AMPK is known to have multiple roles in activating autophagy. For example, the activated AMPK can upregulate ULK1 by phosphorylating ULK1, and the activation of ULK1 recruits ATG proteins to the membrane to initiate the autophagy process [7,8]. AMPK is involved in many metabolic diseases including diabetes mellitus and fatty liver disease [5,9]. For example, AMPK signaling enhances insulin sensitivity and also inhibits pancreatic β cell deaths to prevent the onset of diabetes [10]. In addition, AMPK is regarded as a tumor suppressor in cancer, and as an AMPK activator, metformin is reported to reduce cancer incidence [11]. The activation of AMPK has been found to inhibit cancer progression in various cancer types [11].

Three-dimensional (3D) cell culture methods can be used in various biological or clinical assays because 3D cell culture can have a high degree of biological relevance [12,13]. For example, the response to the anti-cancer drug will differ between 2D cell culture and 3D cell culture, and the response to the anti-cancer drug in vivo will be more relevant to the 3D cell culture system [14,15]. A 3D spheroid cell culture system can easily set up and produce small 3D cell cultures for biological and clinical assay [16,17]. Therefore, a 3D spheroid cell culture system is widely used to examine the efficacy of anti-cancer drugs [18,19]. Three-dimensional spheroid cell culture can be produced using multiple methods, such as the hanging drop method and cell culture on a non-adhesive surface [20,21,22].

Autophagy is closely related to cell survival in 3D cell culture. Recent reports showed that 3D cell culture is more resistant to starvation than 2D cell culture, and autophagy is supposed to be responsible for the survival of cells in 3D cell culture [23]. Moreover, autophagy induction is associated with the enhanced viability of mesenchymal stromal cells in 3D cell culture [24]. Autophagy induction is also known to confer drug resistance to the tumor spheroids [25,26]. In addition, the knockout of autophagy-related genes like ULK1 decreases the tumor cell viability [27]. Reactive Oxygen Species (ROS), including superoxide, are known to induce autophagy, and autophagy induction can enhance cell viability by modulating ROS levels in 3D cell culture [24]. Several studies have explored the role of AMPK in 3D cell culture. A recent study demonstrated that the pharmacological inhibition of an AMPK upstream kinase disrupts the viability of ovarian cancer spheroids [28]. Another study showed that treatment with an AMPK allosteric inhibitor interferes with the growth of breast cancer spheroids [29]. However, a 3D spheroid study using AMPK knockout cells has not yet been conducted.

Previously, we generated AMPK knockout cells using the CRISPR-Cas9 system and examined various roles of AMPK [30]. AMPK knockout impairs autophagic flux and also downregulates the expression of human telomerase [31]. Recently, we demonstrated that AMPK knockout cells are less susceptible to coronavirus infection [32]. Because we examined the activity of AMPK in a 2D culture system, we wanted to examine the role of AMPK in a 3D culture system, which is more biologically relevant. We used the 3D spheroid model to study the role of AMPK and showed that AMPK is required for efficient spheroid formation.

## 2. Materials and Methods

### 2.1. Cell Culture and 3D Spheroid Formation

HEK293T cells were cultured in DMEM (Welgene, Seoul, Republic of Korea) supplemented with 10% fetal bovine serum (FBS, Thermo Fisher Scientific, Waltham, MA, USA) and 1% antibiotic–antimycotic solution (Welgene). AMPK knockout HEK293T cells were generated using the CRISPR-Cas9 method as described previously [30]. For 3D spheroid formation, HEK293T cells were seeded in ultra-low attachment plates (Corning, Kennebunk, ME, USA), and the cells were spun down using a centrifuge (200× *g*, 5 min). The plate was incubated in a cell culture incubator for the indicated time. The morphology of the 3D spheroid was captured with a microscope equipped with a digital camera (Nikon, Tokyo, Japan), and the diameter of each spheroid was examined.

### 2.2. Transmission Electron Microscopy

HEK293T spheroid cells were fixed with 2.5% glutaraldehyde and treated with 1% osmium tetroxide on ice for 2 h. Finally, the spheroid cells were washed with PBS. The spheroids were dehydrated in ethanol and treated with propylene oxide. The spheroids were embedded in an Epon 812 mixture and polymerized in an oven at 70 °C for 1 day. The sections for imaging were acquired from the polymerized blocks. The spheroids samples were collected on grids, counterstained with uranyl acetate and lead citrate, and examined with a Bio-HVEM system (JEM-1400Plus at 120 kV and JEM-1000BEF at 1000 kV, JEOL, Tokyo, Japan) in Korea Basic Science Institute (KBSI).

### 2.3. Reporter Assay and Western Blot

For the reporter assay, cells were seeded in 2D and 3D microplates and transfected with a total of 0.5 μg of DNA per well. The pGL2-2XCLEAR plasmid (Addgene, #81120) was introduced into the cells using Lipofectamine 2000. Luciferase activity was normalized to Renilla luciferase activity. The dual luciferase reporter assay kit was purchased from Promega (Madison, WI, USA).

For Western blot, the cells and spheroids were collected by centrifugation and lysed in the lysis buffer (150 mM NaCl, 50 mM HEPES (pH 7.5), and 1% NP40) containing a protease inhibitor cocktail (Roche, Basel, Switzerland). Sonication was used to lyse 3D spheroid cell clumps. An equal amount of cell lysates was loaded into SDS-PAGE and transferred into the PVDF membrane. The membrane was subjected to Western blot with anti-LC3 antibody (MBL International, Watertown, MA, USA). Chemi-doc obtained the Western blot signal (Bio-Rad, Hercules, CA, USA).

### 2.4. Immunohistochemistry and Immunofluorescence Analysis of 3D Spheroid Cultures

Spheroids were washed in PBS and fixed with 4% paraformaldehyde for 60 min at room temperature. Spheroids were dehydrated in an ethanol gradient, embedded in paraffin, and sectioned at 5 μm thick. Sections were deparaffinized and rehydrated. Paraffin block sections were stained with hematoxylin or used for immunostaining. Endogenous peroxidase was inactivated using 0.5% hydrogen peroxide in PBS, followed by antigen retrieval using citrate buffer (pH 6.0). Sections were then blocked with 10% normal goat serum for 30 min at RT, and then incubated overnight at 4 °C with anti-LC3 or anti-cleaved PARP-1 antibody. A purified normal rabbit IgG was used as a negative control. After washing, sections were incubated for 1 h at RT with a biotinylated goat anti-rabbit secondary antibody (1 μg/mL; Vector Laboratories, Burlingame, CA, USA), followed by a streptavidin–peroxidase conjugate (Invitrogen, Carlsbad, CA, USA) for 10 min. The substrate solution from the Substrate Kit (Vector Laboratories, USA) was added to the spheroid sections, which were then incubated for 10 min at RT. The spheroid sections were washed in water, counterstained with Mayer’s hematoxylin, and mounted with coverslips. Images were captured using a BX53 microscope (Olympus, Tokyo, Japan). For immunostaining, the sections were counterstained with DAPI and mounted in a mounting medium (Vector Laboratory Ltd., Peterborough, UK). Images were acquired using a Carl Zeiss LSM710 confocal microscope (Carl Zeiss, Oberkochen, Germany). Anti-cleaved PARP-1 antibody was purchased from GeneTex (Irvin, CA, USA).

### 2.5. Flow Cytometry

For cell cycle analysis, cells were washed with PBS and fixed with 70% ethanol in ice. After centrifugation, fixed cells were washed and resuspended in PBS containing 10 mg/mL RNase A at 37 °C for 30 min. Cells were stained with propidium iodide (PI) (50 µg/mL; Sigma, St. Louis, MO, USA) in PBS at 37 °C for 1 h in the dark. Samples were analyzed using a flow cytometer (Becton-Dickinson, Mountain View, CA, USA). A total of 10,000 events were collected per sample, and cell cycle distribution was analyzed using Flowing Software 2.5.1 (Turku Center for Biotechnology, Turku, Finland).

### 2.6. Statistical Analysis

The Western blot, spheroid diameter, surface area, and reporter assay results were evaluated with a 2-tailed Student’s *t*-test using Excel software version 2108 (Microsoft, Redmond, WA, USA). A *p*-value of 0.05 was considered significant. The graph shows the mean and standard error.

## 3. Results

### 3.1. Formation of 3D Spheroid Using Ultra-Low Attachment Plate

**Figure 1:**
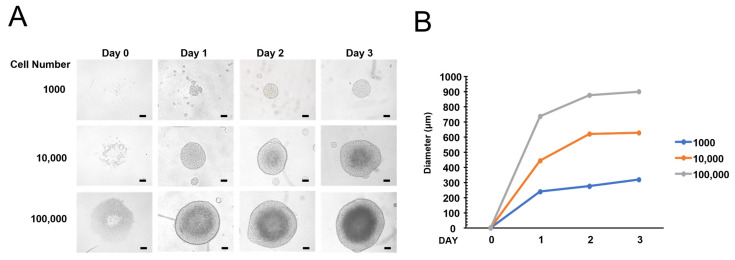
Formation of HEK293T spheroid using ultra-low attachment plate. (**A**) HEK293T cells were seeded at 1000, 10,000, and 100,000 cells/well and incubated for the indicated days. Bars, 100 μm. (**B**) The diameters of the spheroids were measured and are shown in the graph.

An ultra-low attachment plate can be used to form 3D spheroids, and we examined whether HEK293T cells can be used to form 3D spheroids. HEK293T cells have several advantages for monitoring cell signaling, particularly in reporter assays. When 1000, 10,000, and 100,000 cells were seeded for spheroid formation, 10,000 and 100,000 cells formed the spheroids efficiently after 1 day; however, 1000 cells formed spheroids after 2 or 3 days (Figure 1A). Next, we measured the spheroid’s diameter to quantify the growth of spheroids (Figure 1B). We found that the diameter of the spheroids increased over a period of up to 3 days. These results indicate that HEK293T cells can form 3D spheroids efficiently.

### 3.2. AMPK Knockout Delayed Spheroid Growth

**Figure 2:**
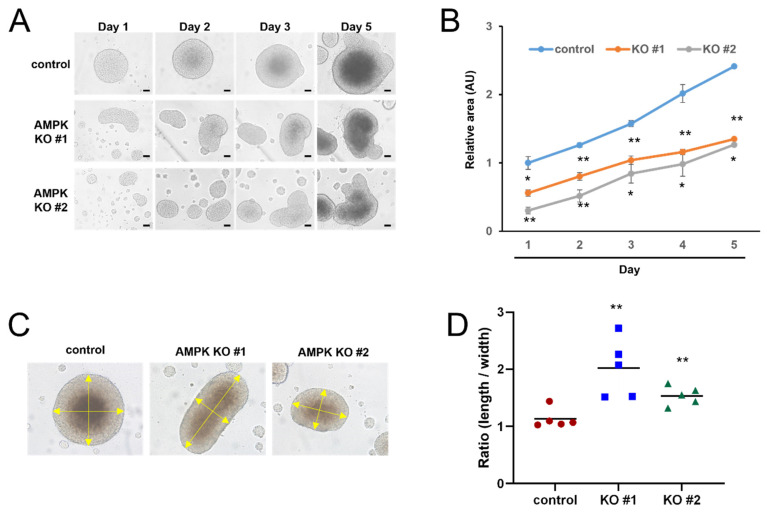
AMPK knockout cells produced oval-shaped spheroids. (**A**) Control HEK293T and AMPK knockout cells were seeded at 10,000 cells/well, and spheroid shapes were captured at the indicated days. Bars, 100 μm. (**B**) The spheroids of AMPK knockout cells were smaller than the spheroids of control cells. The surface area was calculated in each sample and is shown in the graph. N = 5, control vs. AMPK knockout, * *p* < 0.05, ** *p* < 0.01. (**C**) The spheroids of AMPK knockout cells were oval-shaped rather than round-shaped. (**D**) The length-to-width ratio of each spheroid was calculated. N = 5, control vs. AMPK knockout, ** *p* < 0.01.

Previously, we generated AMPK knockout cell lines and demonstrated that AMPK knockout impairs the autophagic flux [30]. AMPK is the major regulator of cellular metabolism, and we examined whether AMPK affects the formation of 3D spheroids. We measured the size of the spheroids using control and AMPK knockout cells. The 3D spheroids were disintegrated after 6 or 7 days, and we measured the size of the spheroid for 5 days (Figure 2A). Control and AMPK knockout cells formed the 3D spheroid, and we attempted to quantify their growth. Because it is impossible to measure the depth of the spheroid, we simply measured the length and width of spheroids and calculated the surface area of the spheroid. Although both the control and AMPK knockout cells formed the spheroid, the sizes of the AMPK KO spheroids were significantly smaller than the control spheroids (Figure 2B). In addition, we found that control cells formed round-shaped spheroids, and AMPK KO cells formed oval-shaped spheroids (Figure 2C). Thus, we calculated the length-to-width ratio of the spheroids. While the length-to-width ratio of control cells was about 1, the ratio of AMPK KO cells was significantly larger than 1 (Figure 2D). These results indicate that AMPK knockout cells formed smaller spheroids than the control cells, and AMPK knockout cells formed oval-shaped spheroids.

### 3.3. The Expression of LC3 Protein Is Increased in AMPK Knockout Cells

**Figure 3:**
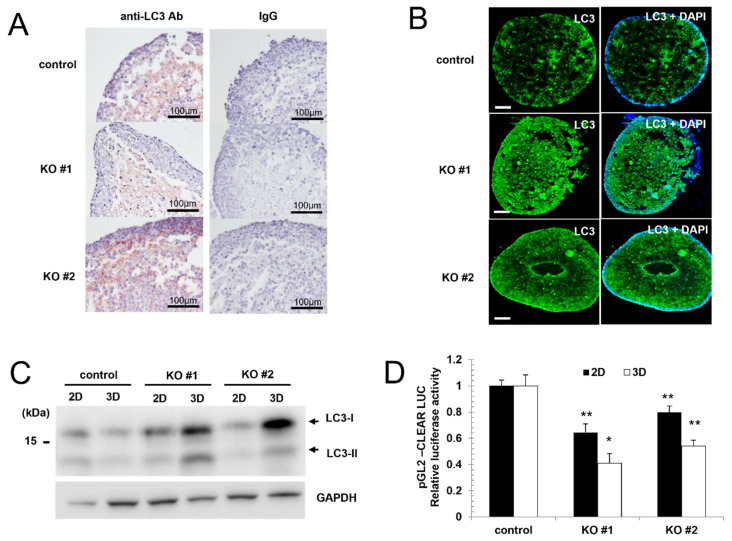
The expressions of autophagy markers are induced in the core of spheroids. (**A**) Control and AMPK knockout spheroid were used to make a paraffin-embedded tissue block before sectioning. Each slide was stained with an anti-LC3 antibody. (**B**) The control cell and AMPK knockout cell spheroid were stained with an anti-LC3 antibody, and the stained cells were observed using a confocal microscope. Bars, 100 μm. (**C**) The expression of LC3 proteins is upregulated in AMPK knockout spheroids. GAPDH was used as a loading control. (**D**) The reported activity of pGL2-CLEAR Luc was reduced in AMPK knockout cells. Control vs. AMPK knockout (N = 3), * *p* < 0.05, ** *p* < 0.01.

Because AMPK plays an important role in the regulation of autophagy, we examined the expression of LC3 protein, an autophagy marker. We examined the expression of LC3 protein in the spheroid using a paraffin-embedded block. When we examined the expression of LC3 protein in the 3D spheroids, we observed a higher expression of LC3 inside the spheroids (Figure 3A,B). Moreover, we also detected a higher expression of LC3 protein in AMPK knockout spheroids using fluorescent microscopy (Figure 3B). The proteins were extracted from the pooled spheroid, and we examined the expression of the LC3 protein to compare the LC3 level. When we examined the expression of LC3 protein in 3D spheroid culture by Western blot, we also found that the level of LC3 expression was increased in AMPK knockout spheroids (Figure 3C). These results collectively indicate that LC3 expression is upregulated in AMPK knockout spheroids.

Transcription factor EB (TFEB) plays an important role in the regulation of lysosomal biogenesis and autophagy [33]. AMPK is known to induce autophagy by regulating TFEB [34,35], and we examined TFEB activity in 3D spheroids. We used the reporter construct containing a coordinated lysosomal expression and regulation (clear) motif [36]. Reporter constructs were transfected into the control and AMPK knockout cells, and we examined the activity of the reporter construct. AMPK knockout cells showed reduced transcriptional activity in 2D culture, and the difference was larger in the 3D spheroid, indicating that AMPK-TFEB signaling is reduced in the 3D spheroid (Figure 3D).

### 3.4. AMPK Knockout Spheroids Have Enlarged Lysosomes

**Figure 4:**
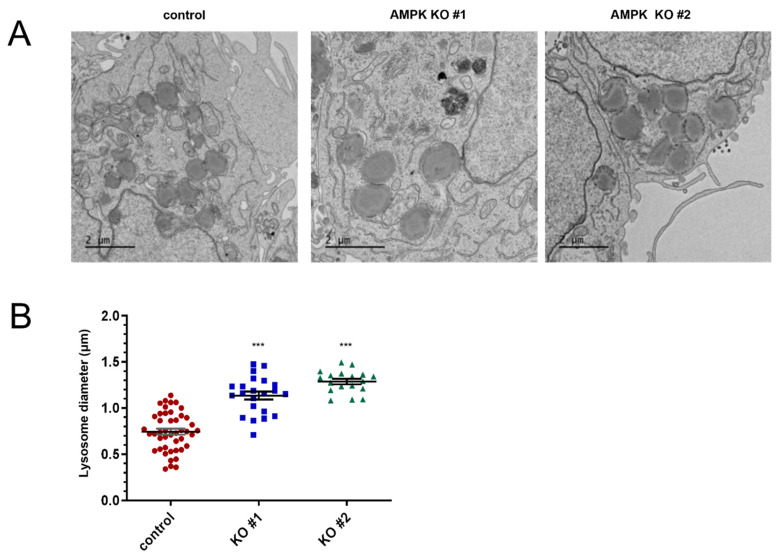
AMPK knockout cells produced larger lysosomes in 3D spheroids. (**A**) Control cells and AMPK knockout cells were analyzed using a transmission electron microscope. (**B**) The size of the lysosome was measured and is shown in the graph. Control vs. AMPK knockout, *** *p* < 0.001.

Because the level of LC3 protein was increased and TFEB activity was reduced in the 3D spheroid culture, we attempted to examine the autophagy-related structure by transmission electron microscopy (TEM). We searched for differences in autophagosome-related structures between the control and AMPK knockout cells. Although we did not see a noticeable difference in the autophagosome structure, we observed the enlarged lysosome in AMPK knockout 3D spheroids (Figure 4A). We measured the size of the lysosome to examine whether the difference was statistically significant. The average diameter of the lysosome in AMPK knockout spheroids is up to twice that of the control spheroids (Figure 4B). When the lysosomal function is impaired, the cells possess enlarged lysosomes [37]. Therefore, these results indicate that AMPK knockout spheroids have larger lysosomes than control cells.

### 3.5. AMPK Knockout Increased the Apoptotic Cells in 3D Spheroid

**Figure 5:**
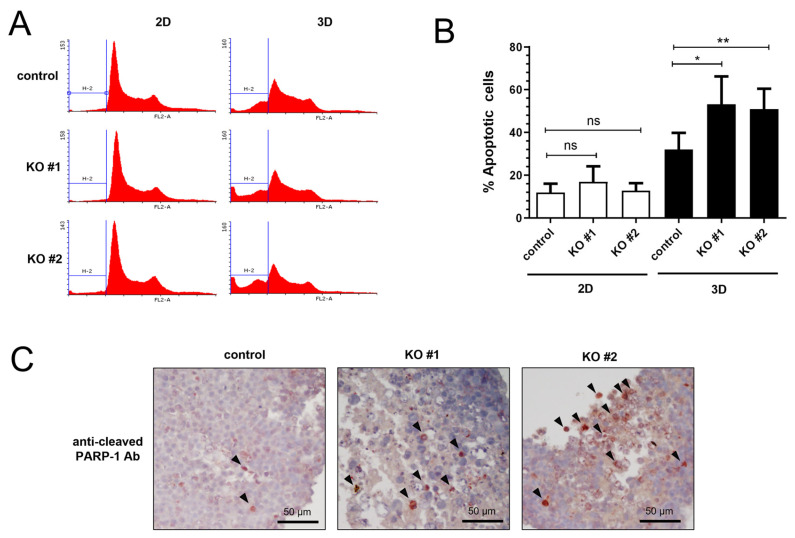
AMPK knockout cells spheroids contain more apoptotic cells than control cells. (**A**) Control HEK293T cells and AMPK knockout cells were used to form the spheroids, and the spheroids were disassembled for flow cytometry analysis. (**B**) The sub-G1 population of each cell was analyzed and is shown in the graph. Control vs. AMPK knockout (N = 3), * *p* < 0.05, ** *p* < 0.01. (**C**) Control and AMPK knockout spheroids were used to make a paraffin-embedded tissue block before sectioning. Each slide was stained with an anti-cleaved PARP-1 antibody. Arrowheads indicate the positively stained cells.

Since the AMPK KO spheroids were smaller than the control spheroids, we examined the cell cycle of the control and AMPK knockout cells. We prepared single cells from 2D cell culture and 3D spheroids and analyzed the cell cycle using flow cytometry. First, we found that the number of apoptotic cells generally increased in 3D spheroid cells (Figure 5A). Moreover, AMPK KO resulted in significantly more apoptotic cells (Figure 5B). The cleavage of poly ADP-ribose polymerase (PARP) is a typical marker of apoptosis induction [38]. We used immunohistochemistry with an anti-cleaved PARP-1 antibody to determine whether these cells undergo apoptotic cell death and found that a subset of cells was positive for anti-cleaved PARP-1 staining (Figure 5C). These results indicate that 3D spheroids have more apoptotic cells than the 2D culture, and AMPK is beneficial for cell survival in 3D spheroids.

## 4. Discussion

In this report, we examined the role of AMPK in forming 3D spheroids. Previously, we generated AMPK knockout cells and used these AMPK knockout cells to examine the role of AMPK in 3D spheroid formation [30,31]. We optimized the experimental condition by seeding different numbers of HEK293T cells in ultra-low attachment plates and tried to produce a 3D spheroid in these conditions (Figure 1). AMPK knockout cells produce significantly smaller spheroid than control cells, suggesting that AMPK is required for efficient spheroid formation (Figure 2). Interestingly, AMPK knockout cells tend to produce oval-shaped spheroids rather than round-shaped spheroids. Because AMPK knockout produced significantly smaller spheroids, we examined the cell cycle. We found that 3D spheroids contain more apoptotic cells than 2D cell culture (Figure 5). In addition, AMPK knockout spheroids contain more apoptotic cells in the spheroids (Figure 5). For this reason, we speculate that AMPK is required for the survival of cells inside spheroids, and AMPK knockout interferes with the survival of inner cells. Therefore, we assume that this is why AMPK knockout tends to form oval-shaped spheroids.

AMPK plays an important role in autophagy regulation. Therefore, AMPK knockout impairs autophagy initiation and progression [39,40]. Here, we also observed that autophagy is impaired in AMPK knockout spheroids. TFEB-dependent transcription using a clear motif is impaired, and we also found that the diameter of the lysosomes is increased in 3D spheroids (Figure 3 and Figure 4). The enlarged lysosomes are found when the lysosomal functions are impaired [41]. When the fusion of lysosomes with autophagosomes is impaired, the enlarged autophagosomes can be formed [37,41]. TFEB is also involved in regulating lysosomes, and the reduced activity of TFEB can result in an enlarged lysosome [42]. Because the lysosomal function is essential to the autophagic flux [43], our results indicate that autophagic flux in AMPK knockout 3D spheroids is impaired. The accumulation of LC3 protein can be more evidence for the impairment of autophagic flux.

Recent reports support that autophagy is required for efficient 3D spheroid formation [24,25]. The knockout of ULK1, an autophagy regulator, is reported to decrease the cell viability of 3D spheroids [27]. In this report, our results suggest that AMPK, an important autophagy regulator, is required for efficient 3D spheroid formation. A previous study reported that the pharmacological inhibition of AMPK decreased the viability of ovarian cancer spheroids; however, STO-609, the inhibitor used in that study, targets calcium/calmodulin-dependent protein kinase kinase beta (CAMKKβ), an upstream kinase of AMPK [28]. Thus, the current findings from AMPK knockout experiments further support the previous results by directly targeting AMPK. Because tumor mass in vivo is more like 3D cell culture rather than 2D cell culture, the inhibition of AMPK will be helpful in improving cancer therapy. Efficient AMPK inhibitors, such as Compound C, are available, and several studies have reported that treatment with Compound C interferes with cancer cell growth [44,45]. Therefore, these inhibitors can be used to improve cancer therapy in combination with other cancer medicines. In this study, we used HEK293T cells to produce 3D spheroids; however, human cancer cell spheroids will be a more suitable model for examining the effect of AMPK inhibition in the context of cancer therapy. Further study will be required to examine the anti-cancer effect of the treatment of AMPK inhibitors with anti-cancer drugs using 3D tumor spheroids. AMPK knockout cells could be a suitable option for confirming the combinatorial effects. In addition, the development of more specific AMPK inhibitors would be desirable to avoid potential side effects caused by interference with other kinases.

## Data Availability

Data are contained within the article.
